# Age‐dependent male mating tactics in a spider mite—A life‐history perspective

**DOI:** 10.1002/ece3.2489

**Published:** 2016-09-22

**Authors:** Yukie Sato, Peter T. Rühr, Helmut Schmitz, Martijn Egas, Alexander Blanke

**Affiliations:** ^1^ Sugadaira Montane Research Center University of Tsukuba Ueda, Nagano Japan; ^2^ Institute for Biodiversity and Ecosystem Dynamics University of Amsterdam Amsterdam The Netherlands; ^3^ Zoologisches Forschungsmuseum Alexander Koenig Zentrum für Molekulare Biodiversitätsforschung Bonn Germany; ^4^ Institute for Zoology University of Bonn Bonn Germany; ^5^ Medical and Biological Engineering Research Group School of Engineering University of Hull Hull UK

**Keywords:** alternative reproductive tactics, male mate competition, residual reproductive value, resource holding potential, *Tetranychus urticae*

## Abstract

Males often fight with rival males for access to females. However, some males display nonfighting tactics such as sneaking, satellite behavior, or female mimicking. When these mating tactics comprise a conditional strategy, they are often thought to be explained by resource holding potential (RHP), that is, nonfighting tactics are displayed by less competitive males who are more likely to lose a fight. The alternative mating tactics, however, can also be explained by life‐history theory, which predicts that young males avoid fighting, regardless of their RHP, if it pays off to wait for future reproduction. Here, we test whether the sneaking tactic displayed by young males of the two‐spotted spider mite can be explained by life‐history theory. We tested whether young sneaker males survive longer than young fighter males after a bout of mild or strong competition with old fighter males. We also investigated whether old males have a more protective outer skin—a possible proxy for RHP—by measuring cuticle hardness and elasticity using nanoindentation. We found that young sneaker males survived longer than young fighter males after mild male competition. This difference was not found after strong male competition, which suggests that induction of sneaking tactic is affected by male density. Hardness and elasticity of the skin did not vary with male age. Given that earlier work could also not detect morphometric differences between fighter and sneaker males, we conclude that there is no apparent increase in RHP with age in the mite and age‐dependent male mating tactics in the mite can be explained only by life‐history theory. Because it is likely that fighting incurs a survival cost, age‐dependent alternative mating tactics may be explained by life‐history theory in many species when reproduction of old males is a significant factor in fitness.

## 1 Introduction

Most males fight with conspecific males for access to females (Andersson, 1994). At the same time, alternative male mating tactics such as sneaking, satellite behavior, and female mimicking have been described for many species (e.g., Brockmann, 2001; Gross, 1996; Radwan, 2009). Generally, if alternative male mating tactics comprise a conditional strategy (Gross, 1996), the mating tactics are often determined by their “resource holding potential” (RHP; Parker, 1974), which can be expressed in terms of body size and energy reserves. The RHP is believed to directly affect the likelihood to win fights. Alternative male mating tactics have often been considered as a “best of a bad job” strategy which allows the less competitive males to produce at least a few offspring (e.g., Brockmann, 2001). However, alternative mating tactics are not only used by inferior males. For example, in the red‐winged blackbird, *Agelaius phoeniceus*, competitively superior males sire offspring in the nests of neighbors (sneaking tactic) while they establish their own territories (conventional, territorial tactic) (Weatherhead & Boag, 1995). Such an alternative mating tactic alongside the conventional tactic by competitive males was also observed in the nesting threespine stickleback, *Gasterosteus aculeatus* (Candolin & Vlieger, 2013; Kemp, 2006). These fishes may display alternative mating tactics even if their RHPs are higher than that of their conspecifics and also even regardless of their RHPs, as long as application of alternative mating tactics increases reproductive success of males.

In the cases of the blackbird and the stickleback, males display two different mating tactics simultaneously to increase their reproductive success further in a breeding season. Thus, from a life‐history perspective, sequential application of different mating tactics can also increase lifetime reproductive success. In a broad array of taxa, young males have been observed to display less aggressiveness and nonfighting mating tactics (Kemp, 2006). In long‐lived animals, a trade‐off between current and future reproductions often exists (Roff, 2002). Male fights are always costly, since males spend time and energy (e.g., Hack, 1997), may get injured, or even die from such fights (e.g., Crespi, 1988; Hamilton, 1979; Reece, Innocent, & West, 2007; Saitō, 1990). If males that employ alternative male mating tactics avoid the risks and costs of fighting and thereby promote their future reproduction, these risks and costs of fights can be considered a resource whose temporal allocation across the male lifetime can be optimized (Kemp, 2006; Kokko, 1997). The evolution of lifetime fighting behavior was investigated theoretically from this life‐history perspective, that is, focusing on the effect of reproductive values (the likelihood of future reproduction; Fisher, 1930). The theoretical analysis showed that the *late‐hawk* strategy (nonaggressive when young but aggressive when old) is evolutionarily stable under a wider range of conditions. For example, the range of benefit and cost of aggressive tactics in which *late‐hawk* strategy evolves was broader than those of the other three strategies, the *hawk* strategy (always aggressive), the *dove* strategy (always nonaggressive), and the *early‐hawk* strategy (aggressive when young but nonaggressive when old) (Kemp, 2006). Young males have higher reproductive values and may have many more opportunities to participate in future matings; therefore, the trade‐off between current and future reproduction is stronger for young males. Ensuring survival and saving their fighting ability for their future reproduction may contribute to maximize their lifetime reproductive success under a broad range of situations.

The theoretical analysis also found that a *late‐hawk* strategy evolves even when the cost and benefit of aggressive tactics are relatively low and high, respectively, if the RHPs increase with age (Kemp, 2006). Therefore, it should be possible to test the importance of RHP on the evolution of lifetime fighting behavior as well. However, RHP is often associated with age. For example, in mammals, birds, and reptiles, males are likely to grow and become larger after sexual maturation. Interestingly, the theoretical work found that the *late‐hawk* strategy can evolve without an increase in RHP, whereas the evolution of an *early‐hawk* strategy (aggressive when young but nonaggressive when old) was predicted only when RHP is expected to decline with age, irrespective whether prior contest participation took place (Kemp, 2006). Testing these predictions requires strictly defined mating environments in order to clearly separate the effect of reproductive values from the effect of RHP.

Recently, in the two‐spotted spider mite (*Tetranychus urticae* Koch), a sneaking and a fighting tactic were described (Sato, Sabelis, Egas, & Faraji, 2013). This mite is a polyphagous herbivore <1 mm in length and a known pest of a variety of agricultural crops (Helle & Sabelis, 1985). In this mite, the first mating virtually ensures paternity of offspring (Boudreaux, 1963; Helle, 1967; Satoh, Yano, & Takafuji, 2001). To become the first mate for females, males display precopulatory guarding behavior: they mount females in the last molting stage before adulthood and try to keep this position by fighting with rival males and chasing them away (hereafter, “fighter males”) (Potter, Wrensch, & Johnston, 1976a, 1976b; Sato et al., 2013). In the male fight, males raise and spread their first pair of legs and strike the opponents. They extend their stylets and try to attach a silk droplet from the stylets to the opponent. Attempts to puncture the opponent's integument with their stylets have been recorded as well (Potter et al., 1976a, 1976b). The male fight is intensive and males can easily get injured or even die (Potter et al., 1976a, 1976b). However, some younger males (up to 30% depending on male density and age; Sato, Sabelis, & Egas, 2014) do not show fighting behavior when challenged, but still succeed in mounting the females (hereafter, “sneaker males”). Furthermore, they are less attacked by other males compared to fighter males even when in the mounting position (Sato et al., 2014). A distinct behavioral change of mating tactics from fighting to sneaking was observed when young fighter males were exposed to old fighter males, but a change of tactics in the opposite direction was rarely observed (Sato et al., 2014).

The two‐spotted spider mite thus represents an optimal model system to investigate the evolutionary stability of the *late‐hawk* strategy from a life‐history perspective. Previous studies have shown that larger males are more likely to win the male fights (Potter et al., 1976a, 1976b). However, after sexual maturation, they do not molt anymore; therefore, body size is not a factor influencing age‐dependent mating tactics of the males. Other morphometric differences between fighter and sneaker males, for example, concerning the stylets were also not detected (Sato et al., 2013). In addition, the males switch their mating tactics depending on male density and the age of competitors (Sato et al., 2014), indicating that males can choose their mating tactics without morphological constraints.

Here, we investigate whether the sneaking tactic may increase the likelihood of future reproduction in the mite, by comparing survival of fighter and sneaker males after experiencing mild or strong male competition. Even though fighter and sneaker males are morphometrically indistinguishable, one factor that may still vary with age is the toughness of the outer skin (cuticle). As in all invertebrate species, the cuticle of an individual mite just after ecdysis is soft and hardens over time. The cuticle of a young male is possibly easy to penetrate by the stylets of rival males. Hence, we investigate whether the toughness of this armor is in fact age‐dependent. Due to the small size of the spider mite, we used nanoindentation, a technique normally used to test the material properties at the micrometer scale. The technique has been validated for a range of hard and soft materials (Klocke & Schmitz, 2012; Mann, 2005; Müller, Olek, Giersig, & Schmitz, 2008; Oliver & Pharr, 1992) and should be able to give an indication about the hardness of a given material, such as the outer cuticle of spider mites at different ages.

## 2 Materials and methods

### 2.1 Mites

Commercially obtained mites (Koppert Biological Systems, The Netherlands) were reared on detached kidney bean leaves, *Phaseolus vulgaris* L., on wet cotton wool in a plastic box under constant climatic conditions (25 ± 1°C, 60 % RH and LD 16:8 hr photoperiod).

To prepare a large number of males, we made use of the fact that spider mites exhibit a haplodiploid sex determination system: females develop from fertilized eggs (2*n*), and males develop from unfertilized eggs (*n*). Twenty females in the final molting stage (teleiochrysalis stage) were randomly collected from the culture, and they were introduced together onto a 5 × 5 cm fragment of bean leaf placed on wet cotton wool in a plastic box. The females were allowed to molt into the adult phase and to lay haploid eggs for a week. The females were removed and the eggs were left to develop into males. The males developed in this separate culture were used for the following experiments.

### 2.2 Male survival

We compared survival rates of young fighter males and sneaker males after male competition. We also considered the intensity of male competition, because males change their mating tactics depending on male density, which can be an index of intensity of male competition (Sato et al., 2014). We carried out experiments implementing “mild” male competition (MMC setup), in which a young fighter or sneaker male was forced to compete with four old males, and experiments implementing “strong” male competition (SMC setup), in which a young fighter or sneaker male was forced to compete with eight old males. As a control, we also investigated the survival rates of young males who were not exposed to any form of male competition. We carried out experiments with the MMC and SMC setups separately. We carried out the control treatment in each setup. By comparing male survival in control males between the setups, we checked that the overall quality of males used in the experiments, as reflected in this life‐history parameter, was the same between these two setups. Details of the process of the experiment were as follows.

Males in the final molting stage were collected from the cultures with a wet fine brush and put individually onto bean leaf disks (1.5 cm in diameter) placed on wet cotton wool in a plastic box. We checked their molting status on a daily basis and obtained young males, that is, adult males that molted <24 hr earlier, and old males, that is, adult males molted at least 4 days earlier. We marked the dorsum of all old males using a very small droplet of water‐based paint (red, pink, blue, green, yellow, or sky blue) to discriminate young males from old males. We introduced five young males onto a kidney bean leaf disk together with a female in the final molting stage in order to induce mounting behavior and determine sneaking or fighting tactic in the young males as follows. After one hour, we checked whether one of the males mounted the female. Then, we identified the mating tactic of that male (fighter or sneaker) with a disturbance test. If the guarding male responded actively and showed a fighting posture toward the male on the fine wet brush, the behavior was classified as fighting. If he did not show any response to the male on the brush, the behavior was classified as sneaking (Sato et al., 2013). The trials in which no males or more than one male mounted the female were discarded. We obtained 53 young sneaker males and 55 young fighters for the MMC setup and 53 young sneaker males and 56 young fighter males for the SMC setup. After mating tactic identification, we removed the other four young males and introduced four (MMC setup) or eight old males (SMC setup) for the competition load. One hour after the introduction of the old males, we checked whether the young male kept the mounting position or one of the old males had taken over the position from the young male. Subsequently, we again recorded the mating tactic of those young males that retained the mounting position, because we know from earlier work (Sato et al., 2014) that some males change their mating tactics under these experimental conditions. We then gently picked up each young male using a wet fine brush and placed it individually onto a new bean leaf disk together with a female in the final molting stage. We checked survival of the male daily and replaced the female and the bean leaf disk every 5 days. In the control treatment, we checked the survival of young males who did not have any experience of male competition (i.e., exposure to a female in the final molting stage but no exposure to a group of young males nor to a group of old males thereafter) but were otherwise treated the same.

We compared the proportions of sneaker and fighter males that retained their mounting position between MMC and SMC setups using Fisher's exact test. We also compared the proportions of sneaker and fighter males in each setup separately using Fisher's exact test on the mating tactics identified before competition with old males. To check male quality difference between MMC and SMC setups, we compared the survival curves of males between control treatments in MMC and SMC using a Cox proportional hazards regression model and multiple comparisons for parametric models with Tukey contrasts. To compare male survival rates between fighter and sneaker males, we constructed a Cox proportional hazards regression model with the mating tactics (fighter or sneaker), outcome of the competition (winner or loser), and the interaction as factors in each setup. In the analyses, we used the mating tactics that males displayed after competition with old males, as one to six males changed their mating tactics. When the interaction did not have a significant effect (α* *= 0.05), the interaction term was removed from the model. The proportional hazards assumption of a Cox regression was checked in each model. The statistics software R ver. 3.2.0 (R development Core Team 2015) and the statistic packages *survival* (Therneau & Grambsch, 2000; Therneau & Thomas, 2015), *MASS* (Venables & Ripley, 2002) and *multcomp* (Hothorn, Bretz, & Westfall, 2008) were used in these analyses.

### 2.3 Measurement of outer skin characteristics

Males in the final molting stage were collected with a fine wet brush and individually placed on bean leaf disks (1.5 cm diameter). Their molting status was checked, and 10–20 males of (1) less than 3 hr, (2) 6–9 hr, (3) 24–27 hr, (4) 3–4 hr, (5) 6–7 days, and (6) more than 10 days after emergence were obtained and put in Bouin liquid in 2.0‐ml microtubes. Five to ten males of each life stage were embedded in samples of Epoxy Resin L. During the first minutes of hardening, the mites were oriented such that the body faced the tip of the resin block in order to ensure proper measurement. The resin blocks were hardened over two days and subsequently ultracut with a 6 mm diamond knife (DIATOME, Switzerland) until the surface of the spider mite was reached. This generated a smooth sample surface ideal for material property measurements.

Mechanical tests were conducted in ambient air in a nanomechanical test system capable of normal loading, as well as undergoing in situ SPM (Tribo‐Scope; Hysitron, Minneapolis, USA). We measured hardness (the resistance to constant deformation of a given material) and Young's modulus (the resistance to a reversible deformation) to characterize the material properties of the cuticle. Indentation tests were performed using a three‐sided Berkovich diamond tip with a total included angle of 142.3° (E‐module 1.141 GPa, Poisson 0.07). An area function covering all contact depths obtained in the measurements was established by indenting in a polymethyl methacrylate test specimen with known hardness and modulus. Nano contact depths ranged from 472 to 2,284 nm. The maximum load during indentation was 3,000 μN, with loading and unloading rates of 600 μN/s, and a 2 s holding time at peak load to compensate for material creep. This procedure was conducted five to 19 times per individual. The measurements were carried out in one male of (1) less than 3 hr, two males of (2) 6–9 hr, two males of (3) 24–27 hr, three males of (4) 3–4 days, two males of (5) 6–7 days, and two males (6) more than 10 days after emergence. Hardness (H) and reduced Young′ s modulus (Er) were both calculated from the unloading portions of the load–displacement curves (Bhushan & Li, 2003; Oliver & Pharr, 1992).

The dependency of H and Er on age was analyzed using linear mixed models with male age as fixed factor and individual as random factor. The effect of fixed factor was tested by *F*‐test and the effect of random factor by likelihood ratio test. The statistics software R ver. 3.2.0 (R Development Core Team, 2015) and the statistic packages *lme4* (Bates et al., 2015) and *lmerTest* (Kuznetsova, Brockhoff, & Christensen, 2015) were used.

## 3 Results and Discussion

**Figure 1:**
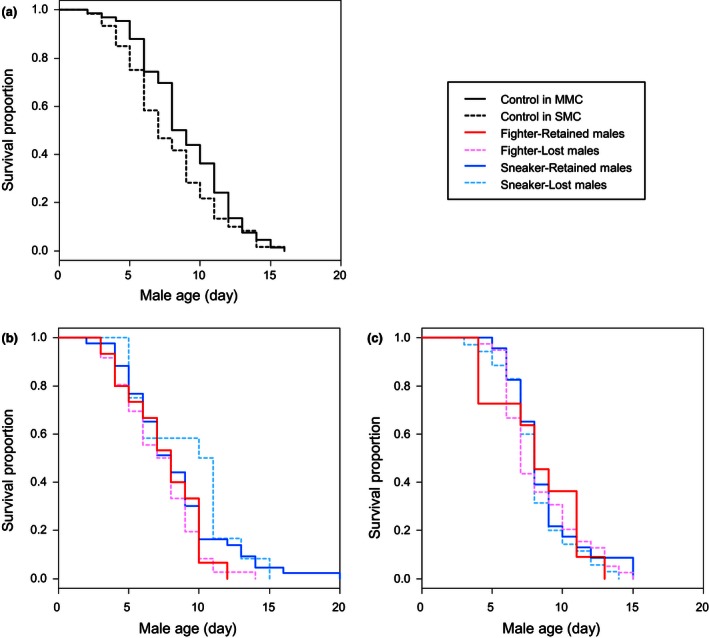
Comparisons in survival curves between control males (a), between fighter and sneaker males that retained and lost their mounting position during mild male competition (MMC) (b), and between fighter and sneaker males that retained and lost their mounting position during strong male competition (SMC) (c). Numbers of replicates are 60 in control males under MMC, 66 in control males under SMC, 53 in fighter males under MMC, 53 in sneaker males under MMC, 51 in fighter males under SMC, and 57 in sneaker males under SMC

Sneaker males survived significantly longer than fighter males after mild male competition (MMC) (Table 1a; Figure 1b). The difference in male survival appears not large with sneaker males living 1.1 days longer on average than fighter males. However, considering that males can mate with several females per day and females in the last molting stage are available constantly especially in large colonies (generations are overlapped and they can always reproduce unless entering diapause; Helle & Sabelis, 1985), the difference in male survival is indeed noteworthy. In addition, we used small leaf disks in the experiment, but generally, the mite lives longer on intact host plants (20 days in the same mite species; Saito, 1979). Therefore, a 1.2 times difference in longevity may result in a large difference in reproductive success.

**Table 1:** Cox proportional hazards regression models of male survival curves in the experiment of mild male competition (MMC) (a) and in the experiment of strong male competition (SMC) (b)

Variable	Coefficient	SE	*z*	*p*
(a)				
Outcome of competition	0.040	0.211	0.191	.848
Male mating tactic	0.442	0.218	2.025	.0428^a^
(b)				
Outcome of competition	−0.159	0.214	−0.744	.457
Male mating tactic	−0.007	0.199	−0.035	.972

a p < .05

(a) Full model: likelihood ratio test: *χ* ^2 ^= 4.43, *df* = 2, *p *=* *.109.

(b) Full model: likelihood ratio test: *χ* ^2 ^= 0.58, *df* = 2, *p *=* *.748.

**Figure 2:**
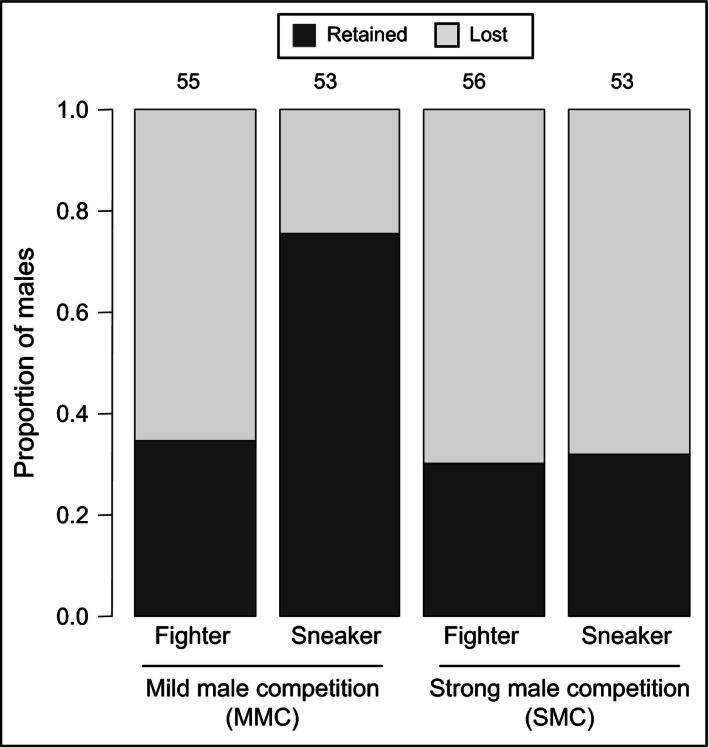
Proportions of sneaker and fighter males that retained or lost their mounting position under mild or strong male competition. Numbers above bars indicate numbers of replicates

We expected a larger difference in survival curves between fighter and sneaker males after strong male mating competition (SMC). However, we did not find such a significant difference (Table 1b; Figure 1c), although we did not detect apparent differences in male quality between MMC and SMC setups (Cox hazard proportional regression model and Tukey contrasts: *z = *1.651, *p *=* *.224; Figure 1a). We predicted that the sneaking tactic always incurs a lower cost to males and thereby a higher lifetime reproductive success, compared to the fighting tactic. However, the cost of performing the sneaking tactic might depend on male density, possibly in relation to the likelihood of being discovered by fighter males. In this study, the proportion of sneaker males retaining the mounting position was significantly lower in SMC than MMC (Fisher's exact test: *p *<* *.001; Figure 2), whereas the proportion of fighter males retaining the mounting position was not significantly different between SMC and MMC (Fisher's exact test: *p *=* *.689; Figure 2). Under MMC, the proportion retaining the mounting position was significantly higher in sneaker males than fighter males (Fisher's exact test: *p *<* *.001; Figure 2), but under SMC, such a difference was not detected (Fisher's exact test: *p *=* *1; Figure 2). Hence, guarding sneaker males are less frequently discovered and attacked by fighter males at lower male densities, but equally frequently at higher male densities. The results are in line with a previous study, where the proportion of sneaker males increased with male density and then decreased again at high male density, that is, there is an optimum density for the expression of sneaker tactic around five males per female (Sato et al., 2014). Hence, there might be an optimum male density for the sneaking tactic to incur the lowest cost of male fight, and under such male density, the sneaking tactic can promote the future reproduction of young males. Therefore, we conclude that our results do support the theoretical prediction of a *late‐hawk* strategy but other factors such as male density affecting the costs and benefits of fighting as well as sneaking are also important to understand the evolution of age‐dependent male mating tactics in the mite.

**Figure 3:**
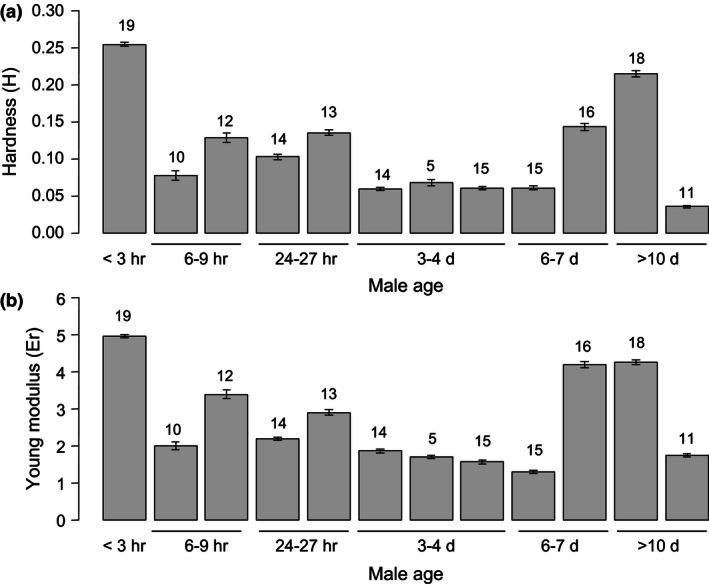
Hardness (a) and Young's modulus (b) of outer skin of variously aged males. Bars indicate averages of the measurements per individual mite, and error bars indicate SE. Numbers above bars indicate numbers of replicate measurements

We found no relationship between skin material characteristics and mite age (Hardness, *F‐*test: *F *=* *1.593, numDF = 5, denDF = 6.02, *p *=* *.43; Figure 3a) (Young's modulus: *F‐*test: *F *=* *1.142, numDF = 5, denDF = 6.04, *p *=* *.43; Figure 3b). In a previous study (Sato et al., 2013), morphometric differences were not detected between fighter and sneaker males. Also, larger males are more likely to win the male fight (Potter et al., 1976a, 1976b). Hence, we have not found evidence that RHP increases with age in the mite males, although the RHP theory is considered as explanation for most conditional male mating strategies (e.g., Brockmann, 2001; Gross, 1996). On the other hand, our results do show differences among individuals in skin hardness and elasticity, not related to the age of these individuals (Hardness, likelihood ratio test: *χ* ^2 ^= 52.49, *df* = 1, *p *<* *.001; Figure 3a) (Young's modulus, likelihood ratio test: *χ* ^2^ = 75.61, *df* = 1, *p *<* *.001; Figure 3b). Earlier work also showed that even among young males, some are fighters and others are sneakers. Hence, skin hardness and elasticity may still relate to the tendency of males to display fighting or sneaking, even if these skin characteristics do not change with age of the males.

Recent studies revealed that social experiences such as male fight and mating with females can be a crucial factor determining age‐dependent male mating tactics (Lee, Head, Carter, & Royle, 2014), because individuals gain experience and information as they age, and experience provides skill acquisition (Sharpe, 2005) and information on their own competitiveness (Fawcett & Johnstone, 2010). To exclude possible effects of social experiences on male mating behavior, we kept the males separately until the experiments started. Several papers, however, reported that spider mites adaptively change their foraging behavior depending on experience (e.g., Choh, Ignacio, Sabelis, & Janssen, 2012; Drukker, Bruin, Jacobs, Kroon, & Sabelis, 2000; Egas, Norde, & Sabelis, 2003; Rahmani, Hoffmann, Walzer, & Schausberger, 2009). Hence, it is possible that males of this mite species also use their experience to adaptively choose their mating tactics, that is, to display sneaking or fighting tactics depending on which maximizes their lifetime reproductive success in the environment they experience. We found the *late‐hawk* strategy in the mite males in the absence of social experience (Sato et al., 2014) and used this situation to test the life‐history theory. However, to fully understand the evolution of alternative male mating tactics in the mite, it will be necessary to evaluate the effect of experience on the alternative male mating tactics.

## Conflict of Interest

None declared.
