# Supplementary figures and images for: Age‐dependent male mating tactics in a spider mite—A life‐history perspective
Source: Ecol Evol. 2016 Sep 22;6(20):7367–74. doi: 10.1002/ece3.2489 (PMC5513254; doi:10.1002/ece3.2489)

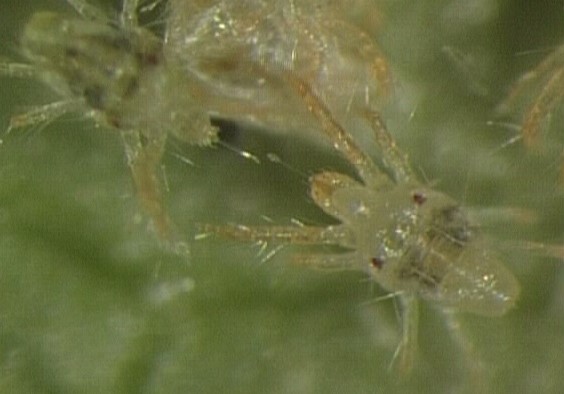

Supplement: Supplementary file 1 [file ECE3-6-7367-s001.jpg]
